# Defined Mathematical Relationships Among Cancer Cells Suggest Modular Growth in Tumor Progression and Highlight Developmental Features Consistent With a Para-Embryonic Nature of Cancer

**DOI:** 10.3389/fcell.2020.00804

**Published:** 2020-08-28

**Authors:** Giovanni Manzo

**Affiliations:** “La Sapienza” University of Rome, Botrugno, Italy

**Keywords:** tumor propagation, tumor hierarchy, cancer stem cell (CSC), tumor sphere, embryo

## Abstract

Several similarities between the embryo development and the cancer process suggest the para-embryonic nature of tumors. Starting from an initial cancer stem cell (i-CSC) as a para-embryonic stem cell (p-ESC), a hierarchic sequence of CSCs (CSC_1_s, CSC_2_s, CSC_3_s) and non-CSCs [cancer progenitor cells (CPCs), cancer differentiated cells (CDCs)] would be generated, mimicking an ectopic rudimentary ontogenesis. Such a proposed heterogeneous cell hierarchy within the tumor structure would suggest a tumor growth model consistent with experimental data reported for mammary tumors. By tabulating the theoretical data according to this model, it is possible to identify defined mathematical relationships between cancer cells (CSCs and non-CSCs) that are surprisingly similar to experimental data. Moreover, starting from this model, it is possible to speculate that, during progression, tumor growth would occur in a modular way that recalls the propagation of tumor spheres *in vitro*. All these considerations favor a comparison among normal blastocysts (as *in vitro* embryos), initial avascular tumors (as *in vivo* abnormal blastocysts) and tumor spheres (as *in vitro* abnormal blastocysts). In conclusion, this work provides further support for the para-embryonic nature of the cancer process, as recently theorized.

## Introduction

It has been theorized recently that several similarities exist between the tumor process and the embryo development (Manzo, 2019). Starting from an initial cancer stem cell (i-CSC/CSC_0_), similar to an ESC without genomic homeostasis (para–ESC, p-ESC), after implantation in a niche, primary self-renewing cancer stem cells (CSC_1_s) would arise, corresponding to epiblast cells. CSC_1_s would then generate secondary proliferating CSCs (CSC_2_s), equivalent to hypoblast cells. CSC_1_s and CSC_2_s, with an epithelial phenotype, would generate, together, tertiary CSCs (CSC_3_s) with a mesenchymal phenotype, corresponding to mesodermal precursors at the primitive streak (PS). Under favorable stereotrophic conditions (normoxia), CSC_3_s would undergo asymmetric proliferation and pre-differentiation into cancer progenitor cells (CPCs) and then into cancer differentiated cells (CDCs), thus giving defined cell heterogeneity and hierarchy (Marjanovic et al., 2013; Singh et al., 2015), mimicking an ectopic rudimentary somito-histo-organogenesis process (Reya et al., 2001; Gibbs, 2009; Ma et al., 2010). In contrast, under unfavorable stereotrophic conditions (hypoxia), CSC_3_s would delaminate and migrate as quiescent micro-metastases, mimicking morphogenetic movements and localizing in metastatic niches (Cabrera et al., 2015; Singh et al., 2015; Yang et al., 2018). Here, specific signals, similar to those occurring in the gastrula inductions, would induce an EMT/MET switch (Thiery et al., 2009; Liu et al., 2014) reverting quiescent CSC_3_s to proliferating CSC_1_s. These cells would be able to generate macro-metastases with the same cell hierarchy as their primary tumors (Marjanovic et al., 2013). Now, I intend to show that the above-proposed tumor hierarchy, from CSCs to CDCs, allows the prediction of a tumor proliferation model that is in strong agreement with some experimental data reported for mammary tumors (Liu et al., 2014). Therefore, it is possible to identify specific mathematical relationships among cancer cells (CCs) occurring in the tumor mass. Moreover, this model suggests that during progression tumor growth might occur in a modular way, which recalls features of tumor spheres and pre-implantation blastocysts (Johnson et al., 2013; Vinnitsky, 2014).

## Cell Heterogeneity, Hierarchy, and Plasticity in Cancer

The tumor bulk consists of several types of cells, encompassing Cancer cells (CCs), stroma cells, endothelial cells, and immune cells (Hanahan and Weinberg, 2011). In many tumors, phenotypic and functional heterogeneity among the various cells exists (Marjanovic et al., 2013; Singh et al., 2015), arising from different factors: endogenous, like genetic (mutations) and epigenetic (miRNA, HLA-G, HIF, TGF-beta, BMP); and exogenous, such as niche contact, microenvironment nutrients, pH, space, chemotherapeutic agents. Currently, three different theories try to explain the cell hierarchy and heterogeneity in tumors: (a) the clonal evolution model, (b) the classical CSC model, and (c) the plastic CSC model (Singh et al., 2015). The clonal evolution model proposes that stochastic accumulating mutational events create raw material for the selection of clones of novel cell populations in the same tumor. Each of these cells would be able to generate metastases with particular features, which are different for other metastases and primary tumors. Since it is generally shown that metastases recapitulate the cell hierarchy of the primary tumor in terms of cell type and percentage (Gupta et al., 2011; Marjanovic et al., 2013; Liu et al., 2014; Cabrera et al., 2015; Singh et al., 2015), the clonal model seems unrealistic. The classical CSC model proposes that tumor heterogeneity arises from CSCs that transit through different states (epithelial and mesenchymal) of stemness and differentiation (CPCs and CDCs) by unidirectional conversion from CSCs to non-CSCs (Singh et al., 2015). This model, where CSCs would be at the apex of the process, might better account for heterogeneity and hierarchy of cells in the same tumor, but it does not account for recent reports showing that non-CSCs might revert to CSCs (Chaffer et al., 2011; Gupta et al., 2011; Kim et al., 2013; Singh et al., 2015; Lu et al., 2020). The plastic CSC model proposes that bidirectional conversions are possible between CSCs and non-CSCs, suggesting that during the tumor process, non-CSCs might be induced into CSCs, thus creating new tumor populations (Chaffer et al., 2011; Kim et al., 2013; Singh et al., 2015; Lu et al., 2020). This model might account for both heterogeneity and hierarchy by plasticity of non-CSCs through a context-dependent behavior influenced by microenvironmental signals. Recently, I suggested that tumor heterogeneity and hierarchy might result from the para-embryonic nature of the i-CSC/CSC_0_ (Manzo, 2019), which, by the reactivation of an intrinsic genic program, would give rise to a sort of ectopic rudimentary somito-histo-organogenesis, tracing in some way that of the tissue of origin (Reya et al., 2001; Gibbs, 2009; Levings et al., 2009; Ma et al., 2010). Here, naturally epithelial, mesenchymal, progenitor, and differentiated tumor cells would be progressively generated (Bradshaw et al., 2016). Such a genic program would also be realized within related macro-metastases, accounting for the fact that, in general, metastatic cell heterogeneity and hierarchy recapitulate those of the primary tumor. On the other hand, stochastic mutations in the genic program of some CSCs or epigenetic and micro-environmental factors would also be responsible for metastases with a cell heterogeneity different from that in the primary tumor. The plasticity of non-CSCs reverting to a CSC state might be made possible by the genetic instability caused by the absence of genomic homeostasis in the i-CSC/CSC_0_ and handed down throughout all its progeny, including CDCs. This instability would allow non-CSCs to be *de novo* reactivated (neo-re-programmed) in their pluripotency gene regulatory network (OCT4, SOX2, NANONG, KLF4, MYC) by endogenous, niche and/or microenvironmental signals, probably in a different way from the original i-CSC/CSC_0_, thus generating new tumor cell populations (Iliopulos et al., 2011; Kim et al., 2013; Cabrera et al., 2015; Singh et al., 2015; Yang et al., 2018; Lu et al., 2020). Depending on its genetic, epigenetic and microenvironment conditions, a tumor cell could thus realize a defined genic program (“inductive gene chain”) that confers specific phenotypic and physio-pathological features, responsible for a peculiar cell heterogeneity and hierarchy.

## Theoretical Proliferation Model in Cancer: The Tumor Growth Module

**FIGURE 1:**
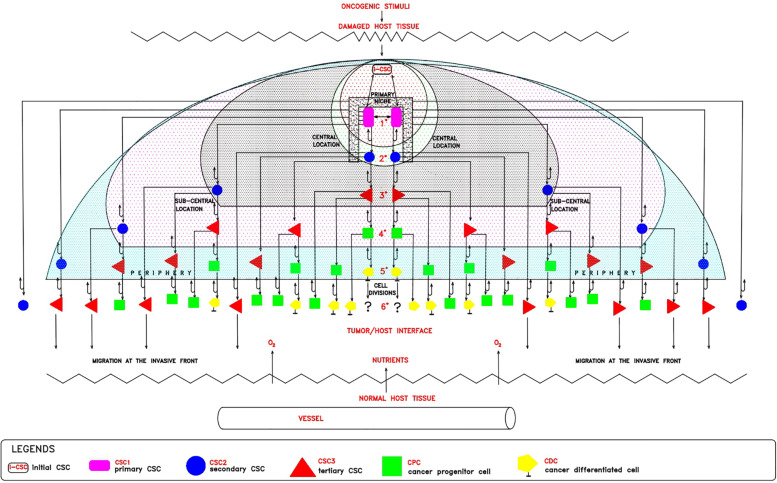
Theoretical tumor growth model: cell hetrogeneity and hierarchy. In a damaged host tissue, the initial cancer stem cell (iCSC/CSC_*o*_, white rectangle_)_ would install itself in a primary niche (black square), where, by expanding symmetrical self-renewal, would generate 2 epithelial CSC_1_s (violet rectangles) located at a central position. Each CSC_1_, by committing asymmetrical self-renewal, would generate 1 maternal CSC_1_, remaining at a central position in the niche, and 1 epithelial committed CSC_2_ (blue circles), located at a peri-niche sub-central position. Each CSC_2_, by asymmetrical autocrine/paracrine division, would generate 1 maternal CSC_2_ and 1 mesenchymal CSC_3_ (red triangles), located in a more peripheral position. After a certain division number, CSC_3_s generated at early stages, would become more external and proximal to the normoxic host tissue, where they proliferate by asymmetrical division, yielding 1 maternal CSC_3_ and 1 pre-differentiated CPC (green squares) located at the border of the process, at the interface between the tumor and the host. CSC_3_s generated at later stages, more internal and thus under hypoxic conditions, would remain quiescent and migrate at the invasive front in search of survival conditions in metastatic niches. Each CPC, by asymmetrical division, would yield 1 maternal CPC and 1 differentiated CDC (yellow pentagons). At this point, the question arises as to whether or not CDCs, differentiated but genetically unstable, could further proliferate.

On the basis of the hypothesized p-ESC nature of the i-CSC/CSC_0_, I propose the following model for the establishment of cell heterogeneity and hierarchy within the tumor histological structure (Figure 1). In a merely theoretical way, considering an i-CSC/CSC_0_ and a niche able to contain only (for simplicity) two CSCs, the following events would occur: (a) allocation of i-CSC/CSC_0_ in a niche, at the apex of the entire process, and subsequent CSC_0_/CSC_1_ transition (Nichols and Smith, 2009); (b) initial expanding symmetrical (Morrison and Kimble, 2006; Norton and Popel, 2014) self-renewal of CSC_1_, yielding two epithelial CSC_1_s anchored to the niche (Niola et al., 2012); (c) committing asymmetrical (Knoblich, 2008; Pattabiraman and Weimberg, 2014) self-renewal of CSC_1_s, each yielding a maternal CSC_1_ at a central position in the niche and a committed epithelial daughter CSC_2_ in a sub-central position at the niche boundaries (Liu et al., 2014; Norton and Popel, 2014); (d) asymmetrical (Knoblich, 2008; Pattabiraman and Weimberg, 2014) autocrine/paracrine proliferation of CSC_2_s, each yielding a maternal epithelial CSC_2_ and (via EMT) a mesenchymal daughter CSC_3_ in a more peripheral position; (e) quiescence of more internal CSC_3_s and their migration externally at the tumor invasive front (Liu et al., 2014; Staneva et al., 2019); (f) asymmetrical (Knoblich, 2008; Norton and Popel, 2014; Pattabiraman and Weimberg, 2014) division of more external CSC_3_s, each yielding a maternal CSC_3_ and a CPC in a more peripheral position of the process (Liu et al., 2014; Staneva et al., 2019); and (g) asymmetrical (Knoblich, 2008; Pattabiraman and Weimberg, 2014) differentiation division of CPCs, each yielding a maternal CPC and a CDC, at the interface with the host normal tissues. Within this proliferation model, CSC_1_s-CSC_2_s-CSC_3_s-CPCs-CDCs would constitute a defined “tumor growth module.” It is possible that such a theoretical proliferation model might account for (1) the various types of CCs present in the bulk of mammary tumors (Liu et al., 2014); (2) the different (epithelial and mesenchymal) CSC phenotypes (ALDH1^+^ CD44^+^ Ki67^+^/hypothetical CSC_1_; ALDH1^+^ CD44^–^ Ki67^+^/hypothetical CSC_2_; ALDH1^–^ CD44^+^ Ki67^–^/hypothetical CSC_3_) detected in mammary tumors (Liu et al., 2014; Manzo, 2019); (3) the hierarchy of the various CSCs and non-CSCs present in a tumor (Liu et al., 2014); (4) the histological tumor structure, where CSCs would naturally remain internal, surrounded by more differentiated tumor cells (Liu et al., 2014; Singh et al., 2015); and (5) the position of CSC_3_s generated at early divisions, which would become progressively more external and proximal to the normoxic host tissues, where favorable micro-environmental conditions (space, oxygen, nutrients, pH) exist (Figure 1). Here, they could undergo EMT/MET switch, subsequent asymmetric division and differentiation in CPCs and then in CDCs, thus generating a growth module with a defined cell hierarchy, responsible for a peripheral finger-like morphology (Norton and Popel, 2014). With regards to the CDCs, the question arises of whether or not they are still proliferating: in general, proliferation and differentiation are mutually exclusive, as also it occurs in CCs (Ruijtenberg and van den Heuvel, 2016). However, coincident occurrence of cell division and a differentiated state have also been reported in CCs (Sage et al., 2005; Ajioka et al., 2007); moreover, the eventual occurrence of dividing pre-differentiated CCs must be considered. In contrast, CSC_3_s generated at later divisions would remain more internal and thus under unfavorable hypoxic conditions. Consequently, in an attempt to survive, they would migrate externally in spatially coordinated migration patterns (Thiery et al., 2009; Tiwari et al., 2012; Staneva et al., 2019), at the interface with normal vascularized host tissues, where better stereo-trophic conditions exist, thus creating an invasive front. Here, they could install in metastatic niches as dormant CSC_3_s by EMT signals (WNT, TGFb) and eventually revert to self-renewing CSC_1_s by MET signals (BMP, LIF) (Thiery et al., 2009; Liu et al., 2014; Grosse-Wilde et al., 2015).

## Modular Growth in Avascular Tumors

**FIGURE 2:**
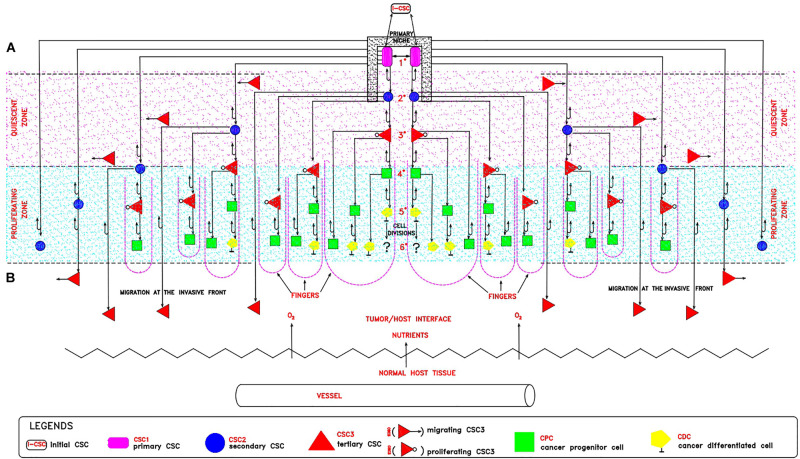
Theoretical tumor growth module: a cord-finger structure. In a tumor growth module, it is possible to distinguish: **(A)** An internal hypoxic zone (pink color) where quiescent CSC_3_s lie, generated at later divisions and migrating externally in spatially coordinated patterns, toward the tumor/host interface, endowed with more favorable stereo-trophic conditions, thus creating an invasive front. Here, they could install in metastatic niches as dormant CSC_3_s by EMT environmental signals (WNT, TGFb) and eventually revert to self-renewing CSC_1_s by MET signals (BMP, LIF). **(B)** An external normoxic zone (light-blue color), where CSC_3_s lie, generated at earlier divisions and thus more proximal to the tumor/host interface, where favorable micro-environmental conditions (space, oxygen, nutrients, pH) exist. Here, these cells could undergo EMT/MET switch, subsequent asymmetric division and differentiation in CPCs and then in CDCs, thus generating a growth module with a defined cell hierarchy, responsible for a peripheral finger-like morphology (violet broken lines). With regards to the CDCs, the question arises of whether or not they are still proliferating.

**FIGURE 3:**
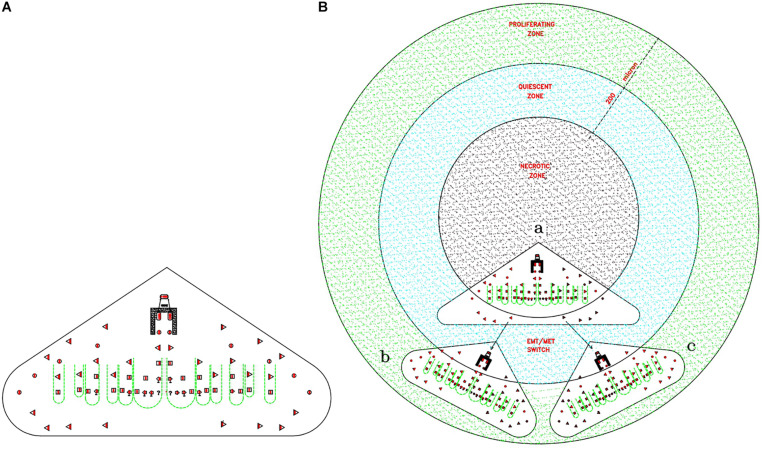
Theoretical relationships between tumor gowth modules and avascular tumors. **(A)** Cord-finger structure of a tumor growth module. This image is directly extrapolated from the Figure 2B. **(B)** Theoretical modular growth in an avascular tumor: **(a)** Initial tumor growth module, located in the central zone, the future necrotic zone (black color). **(b)** and **(c)** Secondary tumor growth modules, arising from migrating CSC_3_s, seeding via EMT/MET switch in surrounding niches, located in the sub-central zone, the future quiescent zone (blue color). Since nutrient diffusion limits are about 200 microns, the primary module comes to lie in the necrotic zone and dies, whereas the later modules located in the quiescent/proliferating zones (blue/green zones) grow, conferring a peripheral finger morphology on the structure. This structure could grow beyond defined limits only if its vascularization occurs, able to supply the necessary nutrients.

**FIGURE 4:**
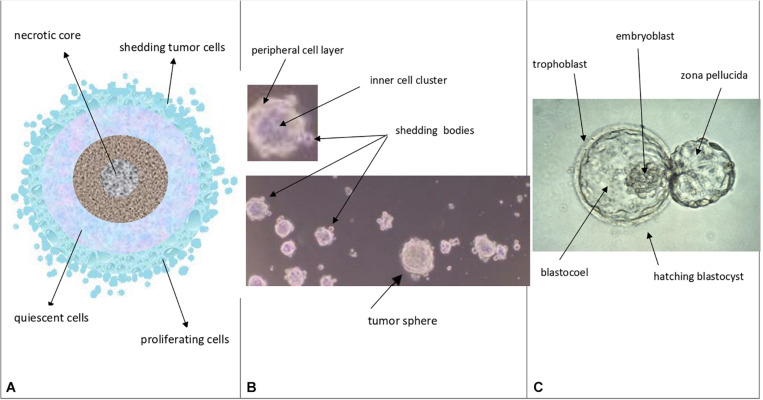
Theoretical comparison among avascular tumors **(A)**, tumor spheres **(B)**, and normal blastocysts **(C)** A. Avascular tumors (as *in vivo* abnormal blastocysts): external proliferating cell layers (light-blue color), middle quiescent cells (gray color) and necrotic core cells (dark colors) are depicted. B. Tumor spheres (as *in vitro* abnormal blastocysts): a layered cell distribution, like in avascular tumors, a peripheral cell layer, similar to the blastocyst trophectoderm, and an inner cell cluster, similar to the blastocyst ICM can be observed; shedding bodies from the sphere surfaces can also be noted (modified and adapted from Bond et al., Plos One. 2013). C. Normal hatching blastocysts (as *in vitro* embryos): trophectoderm, ICM (embryoblast) and blastocoel cavity are indicated, together to the “zona pellucida” (modified from: Human blastocyst hatching. Credit: K. Hardy. CC BY).

The proposed proliferation model would generate tumor growth modules (CSC_1_-CSC_2_-CSC_3_-CPC-CDC) that might be at the basis of and account for the structure and features of the avascular tumor bulk. In particular, mesenchymal CSC_3_s generated early in a tumor growth module would lie in favorable stereotrophic conditions, so they could proliferate, yielding a progeny of CPCs and then CDCs. This progeny could form a hierarchic histological structure that might appear as growth-cordfingers (Norton and Popel, 2014; Figure 2). On the other hand, mesenchymal CSC_3_s generated later within a growth module would lie in unfavorable stereotrophic conditions, so they would be induced to migrate externally for survival. If they find a new niche, they would self-seed (Norton and Popel, 2014) and, by specific signals, undergo EMT/MET switch, becoming self-renewing CSC_1_s able to generate new tumor modules. In such a way, tumor growth could occur by reiterated production of defined cell modules, generating a spherical avascular mass. This might expand until it reaches a diameter of approximately 400 microns, since diffusion and the supply of nutrients and oxygen at the core cells is not possible beyond about 200 microns (Hamilton and Rath, 2019). Assuming for the module cells a middle diameter of about 15 microns, this fact would imply that an avascular tumor bulk might contain about 13 tumor cell layers. Beyond this limit, tumor avascular growth could occur only externally with a simultaneous death of core cells. In such a way, an advanced avascular tumor mass could be a sphere made of (a) an anoxic central zone with necrotic tumor cells, presumably the earlier tumor modules; (b) a sub-central hypoxic zone with the later generated quiescent CSC_3_s that try to migrate externally (Staneva et al., 2019) in search of niches to self-seed around or for metastasizing elsewhere (Norton and Popel, 2014); and (c) a peripheral normoxic zone with the earlier generated proliferating CSC_3_s and their numerous progeny of CPCs and CDCs, resulting together in a cord-finger morphology (Norton and Popel, 2014; Figures 2, 3). Thus, this tumor proliferation model would generate structures that appear to be very similar to real initial avascular tumors and multicellular tumor spheroids (MCTS) (Millard et al., 2017; Hamilton and Rath, 2019; Scientific Reports and Nature Research, 2019; Figure 4).

## Mathematical Relationships Among Cancer Cells

In the model proposed in Figure 1 it is possible to detect numerical relationships among all the CC typologies in a tumor, which surprisingly agree with experimental data shown in a study on 45 primary breast tumors (Liu et al., 2014). By tabulating the theoretical data proposed in Figure 1, it is possible to find well-defined mathematical relationships between CSCs (CSC_1_s, CSC_2_s, CSC_3_s) and non-CSCs (CPCs, CDCs) at each (n) cell division. Starting from a hypothetical low and stable number (two, for simplicity) of CSC_1_s in a niche, for each (n) division, it is possible to define the following relationships (Table 1):

**TABLE 1:** Theoretical mathematical relationships among cancer cells in tumors.

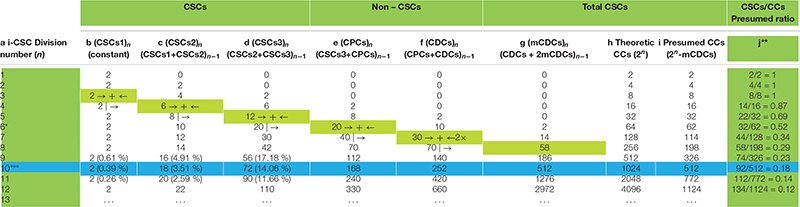

Tabular representation of what is proposed in Figure 1. Theoretical mathematical relationships among CCs in a tumor process could be obtained for each cell typology (CSCs and non-CSCs), as reported in the formulae at the top of Table 1. These formulae can be obtained following the sequence of arrows, as shown. Regarding the total number of CCs, a discrepancy between theoretical (column h) and presumed (column i) values is the result of missing CDCs (column g) arising at the sixth division (*) and increasing progressively in subsequent divisions. The mCDCs might be a natural occurrence if CDCs cannot proliferate, whereas they might be the result of apoptotic death if CDCs are still proliferating; however, in this case, it would not be explainable how a mathematical model might foresee it. The data in Table 1 allow us to determine the presumed ratio between CSCs and total CCs (**), progressively decreasing during tumor progression, as experimentally detected in mammary tumors. Note the high convergence between presumed and experimental CSC percentages (about 18%) at the *n* = 10 division number (***).

$$\begin{array}{ccc} {\left({\mathrm{CSC}}_{1}\,s\right)}_{n}=2\; & {\left({\mathrm{CSC}}_{2}\,s\right)}_{n}={\left({\mathrm{CSC}}_{1}\,s+{\mathrm{CSC}}_{2}\,s\right)}_{n-1}\; & \\ & {\left({\mathrm{CSC}}_{3}\,s\right)}_{n}={\left({\mathrm{CSC}}_{2}\,s+{\mathrm{CSC}}_{3}\,s\right)}_{n-1}\; & \\ & {\left(\mathrm{CPCs}\right)}_{n}={\left({\mathrm{CSC}}_{3}\,s+\mathrm{CPCs}\right)}_{n-1}\; & \\ & {\left(\mathrm{CDCs}\right)}_{n}={\left(\mathrm{CPCs}+\mathrm{CDCs}\right)}_{n-1}\; & \end{array}$$

These relationships theoretically allow us to know, at each (n) division, a presumed total CC number as a sum of the number of each cell typology. In particular, it might be noted that (a) for values of (*n*) from 0 to 5, the total CC number is expressed as a numerical doubling (2^*n*^) (Table 1h,i) (b) from a value (*n*) of 6, the total CC number decreases progressively compared with (2^*n*^) (Table 1h,i), because of missing CDCs (mCDCs), in a quantity expressed by the relationship (Table 1g):

$${\left(\mathrm{mCDCs}\right)}_{n}={\left(\mathrm{CDCs}+2\times \mathrm{mCDCs}\right)}_{n-1}$$

(c) at a value (*n*) of 10, the presumed CC number is exactly half of (2^*n*^), at a value (*n*) of 11, about one third, and at a value (*n*) of 12, a little more than one fourth (Table 1h,i). Thus, the total CC number appears to become progressively more self-limiting; nevertheless, the CSC quantity decreases gradually compared with the total CCs, according to the experimental ratio reported in the literature (Table 1j).

(d) At a value (*n*) of 10, the percentages of CSC_1_s, CSC_2_s, and CSC_3_s result, respectively, in 0.39, 3.51, and 14.06%, with a total of 17.96% CSCs. Surprisingly, these theoretical data, concerning a single niche, are strongly similar to the experimental data found in the mammary tumor mass, namely: 0.084% for ALDH1^+^ CD44^+^ Ki67^+^ CSCs (hypothetical CSC_1_s); 5.54% for ALDH1^+^ CD44^–^ Ki67^+^ CSCs (hypothetical CSC_2_s); 12.87% for ALDH1^–^ CD44^+^ Ki67^–^ CSCs (hypothetical CSC_3_s); and 18.494% for total CSCs (Liu et al., 2014). These similar (presumed/experimental) percentages (about 18%) also occur starting from a niche with a different (3, 4,…) initial CSC_1_ number. Inside these percentages, the discrepancy for CSC1s (0.39 to 0.084%, about 5 to 1) and CSC2s (3.51 to 5.54%, about 1 to 2) might be due to the fact that the proliferation rate for a single theoretical niche is assumed as defined, while in the tumor bulk many niches could have asynchronous growth and a variable proliferation rate. Moreover, in a computational model, stem cell percentages have been found to be between 0.2 and 15%, depending on the simulation parameters (Norton and Popel, 2014). These similarities might thus indicate a true correspondence between ALDH1^+^ CD44^+^ Ki67^+^CSCs and CSC_1_s, ALDH1^+^ CD44^–^ Ki67^+^CSCs and CSC_2_s, ALDH1^–^ CD44^+^ Ki67^–^ CSCs and CSC_3_s, and, consequently, a possible real existence of the hypothesized CSC_1_s, CSC_2_s, and CSC_3_s (Manzo, 2019).

## Similarities Among Avascular Tumors, Tumor Spheres, and Blastocysts

Notably, at (*n*) = 10, many important events seem to occur, as described above. At (*n*) > 10, the correspondence (about 18%) between presumed (17.96%) and experimental (18.494%) CSCs for a single niche tends to diminish progressively. Since the experimental data refer to tumor bulks, certainly with more than 10 cell divisions, it would be possible to question how this correspondence might be conserved in the tumor mass. I therefore hypothesize that it might occur through the proposed “modular growth,” which is able to maintain these percentages throughout tumor progression. In particular, this might be possible if, as proposed earlier, CSC_1_s-CSC_2_s-CSC_3_s-CPCs-CDCs together constituted a tumor growth module (Manzo, 2019; Figure 2). This would self-generate after about 10 division cycles, when the cell number would become presumably too large to survive under unfavorable stereo-trophic conditions (Hamilton and Rath, 2019). For this reason, some CSC_3_s would be induced to delaminate, migrate, and localize in new local or distant niches, where, after EMT/MET switch, they would revert to CSC_1_s (O’Brien et al., 2012; Liu et al., 2014; Grosse-Wilde et al., 2015; Yang et al., 2018; Manzo, 2019) and repeat the modular growth process, thus generating structures with a defined cell heterogeneity and hierarchy (Knoblich, 2008; Johnson et al., 2013; Vinnitsky, 2014). A modular growth process appears to occur also when CSCs cultured *in vitro* under defined conditions form solid, round cellular structures with a diameter of about 50–250 microns, named tumor spheres, through joining of smaller aggregates (spheroids), similar to -single tumor modules (Hamilton and Rath, 2019). Spheroids are also found *in vivo*, as circulating tumor clusters, in ascitic fluid of ovarian cancer and pleural effusions of lung cancers, arising by collective detachment from the tumor bulk (Hamilton and Rath, 2019). These spheroids have a smaller size without the hypoxia and necrotic regions observed in larger 3D structures (Hamilton and Rath, 2019). Tumor spheres are enriched in CSCs, but they also contain non-CSCs, less or more differentiated (CPCs, CDCs) (Cao et al., 2011; Johnson et al., 2013; Liu et al., 2013). The CSCs are endowed with persistent self-renewal, stemness gene expression, high invasiveness, increased tumorigenic potential, and chemo-resistance (Cao et al., 2011; Liu et al., 2013). In such CSCs, expression of NANOG, OCT4, and SOX2 is present, as well as that of ALDH1 and KLF4 (epithelial markers) and CD44 (mesenchymal marker) (Liu et al., 2013). Tumor spheres are tridimensional and mimic the micro-environmental conditions and growth of real tumors. Tumor-sphere cultivation is widely used to analyze the self-renewal capability of CSCs and to enrich these cells from bulk CCs, thus providing a reliable platform for screening potential anti-CSC agents (Knoblich, 2008; Nunes et al., 2018). Large spheroids (400–500 microns in diameter) display a layered cell distribution, also observed in solid avascular tumors: the outer layers are enriched with highly proliferating cells, the middle zone exhibits quiescent cells, and the core contains necrotic cells and acellular regions with hypoxia and nutrient depletion (Norton and Popel, 2014; Galateanu et al., 2016; Millard et al., 2017). Very large tumor spheroids can reach 650 microns in diameter (Zanoni et al., 2016). Morphologically, tumor spheres appear to be defined by a cell layer that resembles the trophectoderm in blastocysts and a cluster of inner cells that resembles the ICM, just like in a preimplantation blastocyst (Johnson et al., 2013; Vinnitsky, 2014) (Figures 4B,C). I suggest that tumor spheres could be an artificial condition mimicking *in vitro* the natural conditions of normal pre-implantation blastocysts (Cao et al., 2011; Vinnitsky, 2014; Nunes et al., 2018), as well as those of *in vivo* avascular tumors (Figures 4A–C and Table 2). Thus, I hypothesize that tumor spheres might be a sort of artificial rudimentary (abnormal) blastocysts which, cultured *in vitro* onto ultralow attachment surfaces in the absence of implantation conditions, display a modular growth behavior similar to that of avascular tumors *in vivo* (Vinnitsky, 2014). This modular growth would also be confirmed by the images of small “shedding” structures, similar to single tumor modules, recently shown on the tumor-sphere surface and released in the surrounding micro-environment (Johnson et al., 2013; Hamilton and Rath, 2019; Figure 4B). The release of such structures resembles and could reflect in some way the “hatching” phenomenon of expanded pre-implantation blastocysts, by which these emerge from the zona pellucida to acquire a condition fit for subsequent implantation (Hardy et al., 1989; Figure 4C). Larger tumor spheres could maintain their state by inducing the release of cells exceeding a cell number (250–280) which would be optimal for eventual implantation. In the absence of micro-environmental conditions that favor implantation, normal blastocysts in the uterus die, while tumor spheres with defined *in vitro* conditions survive and spread, producing shedding growth modules. These would be presumably similar to *in vivo* initial avascular tumors (Vinnitsky, 2014), which could survive as such (dormant) in the absence of suitable implantation conditions or progress in the presence of such conditions. Multicellular tumor spheroid models closely mimic small avascular tumors *in vivo*, with the presence of proliferative cells (about 40%) surrounding quiescent cells and a necrotic core, and with similar gradients of oxygen, pH, and nutrients (Millard et al., 2017; Hamilton and Rath, 2019) (Figure 4B). It has been proposed that tumor spheres fulfill the precondition for a protected niche for dormant tumor cells as an hypoxic niche protected by the outer layers, which exhibit continuous shedding of tumor cells and fragments (Johnson et al., 2013; Hamilton and Rath, 2019).

**TABLE 2:** Similar features among avascular tumors, tumor spheres and preimplantation blastocysts.

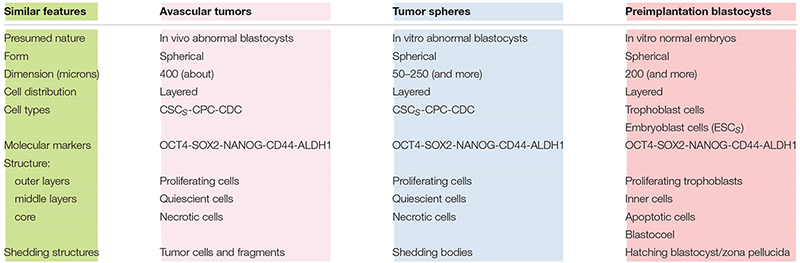

*The major similarities among avascular tumors, tumor spheres and preimplantation blastocysts are summarized and pointed out. This table can also supply some information related to the Figure 4.*

## Discussion and Conclusion

The hypothesis that oncogenesis might be a sort of ectopic rudimentary ontogenesis (Manzo, 2019) would permit us to formulate some considerations and potential explanations for several phenomena: a-Tumor cell heterogeneity and hierarchy, similar in primary and metastatic tumors, might be a natural consequence of the developmental genic program of a de-re-programmed i-CSC/CSC_0_ endowed with para-embryonic features (p-ESC) (Manzo, 2019). b-The plasticity of non-CSCs in the CSC conversion might be made possible by the early genomic instability of the i-CSC/CSC_0_, handed down throughout all its progeny. Therefore, thanks to this condition, a non-CSC could be newly re-programmed in CSC by intrinsic and/or extrinsic signals, eventually also in a different way to the original i-CSC, thus potentially giving rise to a new tumor cell population, with new co-existent heterogeneity and hierarchy arising in the same primary tumor. c-The proposed cell hierarchy model (Figure 1) might account for the global tumor structure shown in mammary tumors (Liu et al., 2014), namely the distinction between CSCs and non-CSCs, the different detected CSC phenotypes, the reciprocal allocation of the different CSCs in the tumor mass, the internal position of CSCs to the external position of the non-CSCs. d-Tabulation of the above proposed cell hierarchy model (Figure 1) permits the elaboration of well-defined formulae for calculating the presumed number of each CC typology and, consequently, the presumed total number of CCs and the CSCs/CCs ratio after (*n*) cell division (Table 1j). This presumed ratio clearly appears to decrease progressively, in agreement with the experimental data reported in the literature. e-However, the presumed total number of CCs seems to be self-limiting for the occurrence of mCDCs. mCDCs could be the result of a lack of further proliferation of CDCs; but, if CDCs were still proliferating, this fact could be due to a natural apoptotic cell death, similarly to what occurs in embryos throughout ontogenesis (Hardy et al., 1989) and in multicellular spheroids (Nunes et al., 2018). In the embryo, widespread cell death by apoptosis in both TE and ICM normally occurs, increasing substantially by about day 7 (Hardy et al., 1989), namely from the 6/7° “one per day” division. Surprisingly, in Table 1, the onset of mCDC occurred just by the 6°cell division and then increased progressively. f-The major indication, resulting from Table 1, is the surprising similarity between the presumed and experimental percentage values for CSCs (ALDH1^+^ CD44^+^ Ki67^+^/CSC_1_; ALDH1^+^ CD44^–^ Ki67^+^/CSC_2_, ALDH1^–^ CD44^+^ Ki67^–^/CSC_3)_, totaling approximately 18% for mammary tumors. g-Such a quantitative correspondence (about 18%) for CSCs could not be a simple coincidence and, if so, constitute a strong indication for the real existence of CSC_1_s, CSC_2_s, and CSC_3_s (Liu et al., 2014; Manzo, 2019). h-CSC1-CSC_2_s-CSC_3_s-CPCs-CDCs, together, could constitute a real tumor progression module that determines modular growth able to maintain a substantially constant ratio of about 18% for CSCs in the tumor mass, as detected in mammary tumors (Liu et al., 2014). In conclusion, I believe this work might contain and supply further indications sustaining the para-embryonic nature of the cancer process, as recently theorized (Manzo, 2019).

## Author Contributions

GM conceived the theory and wrote the manuscript.

## Conflict of Interest

The author declares that the research was conducted in the absence of any commercial or financial relationships that could be construed as a potential conflict of interest.
